# On the significance of germline cytogenetic rearrangements at *MYCN locus* in neuroblastoma

**DOI:** 10.1186/1755-8166-6-43

**Published:** 2013-10-16

**Authors:** Beata S Lipska, Magdalena Koczkowska, Jolanta Wierzba, Anna Ploszynska, Mariola Iliszko, Ewa Izycka-Swieszewska, Elzbieta Adamkiewicz-Drozynska, Janusz Limon

**Affiliations:** 1 Department of Biology and Genetics, Medical University of Gdansk, Debinki 1str, 80211 Gdansk, Poland; 2 Department of General Nursery, Medical University of Gdansk, Debinki 7str, 80211 Gdansk, Poland; 3 Department of Pediatrics, Hematology and Oncology, Medical University of Gdansk, Debinki 7str, 80211 Gdansk, Poland; 4 Laboratory of Pathology and Neuropathology, Debinki 1str, 80211 Gdansk, Poland

**Keywords:** Neuroblastoma, *MYCN*, FRA2C, Partial 2p trisomy

## Abstract

**Background:**

*MYCN* oncogene amplification is the most important prognostic factor in neuroblastoma. 25% neuroblastoma tumors have somatic amplifications at this *locus* but little is known about its constitutional aberrations and their potential role in carcinogenesis. Here, we have performed an array-CGH and qPCR characterization of two patients with constitutional partial 2p trisomy including *MYCN* genomic region.

**Results:**

One of the patients had congenital neuroblastoma and showed presence of minute areas of gains and losses within the common fragile site FRA2C at 2p24 encompassing *MYCN*. The link between 2p24 germline rearrangements and neuroblastoma development was reassessed by reviewing similar cases in the literature.

**Conclusions:**

It appears that constitutional rearrangements involving chromosome 2p24 may play role in NB development.

## Background

Congenital chromosomal aberrations have been of help in identification of causative tumor suppressor gene (TSG) in oncology, to name just two most notorious: *WT1* at 11p13.1 and *RB1* at 13q14.1. Since constitutional deletions and translocations are often the first indicators of the presence of TSGs, the pursue for patients with neuroblastoma (NB) and a concomitant constitutional genomic disorder has started. Disappointingly, so far only a dozen of cases with a germinal chromosomal aberration have been reported to develop NB (Additional file 1: Table S1). Of these, only two regions were found to be affected repeatedly: a loss at chromosome 1p36 and a gain of chromosome 2p. Chromosome 1p36 is known to be deleted in ca. 30% of primary NB tumors, being one of the most common somatic cytogenetic abnormalities of recognizable prognostic significance [1,2]. Accordingly, it has been proposed as the putative *locus* for NB TSG, however so far no major NB predisposition gene has been identified in this region [3].

Conversely, with respect to chromosome 2p two genes involved in NB pathogenesis came into play. Germinal activating mutation in *ALK*, an oncogene lying at chromosome 2p23 has been recognized responsible for most of the familial NB cases, while its somatic mutations are present in 8% of sporadic tumors [4]. Then again, 2p24.3 is the *locus* of *MYCN* gene, amplification of which is considered the most important factor of poor prognosis in NB [5,6]. Since no clear correlation between constitutional rearrangements involving chromosome 2p and the role of *ALK* and/or *MYCN* in early stages of NB pathogenesis has been established, in the current study we have evaluated our two cases and reviewed all reported NB with germinal cytogenetic rearrangements involving chromosome 2p. We used array-CGH and qPCR techniques for further delineation the minimal overlapping region, identification of the causative genes and their role in NB pathogenesis.

## Results

Two patients with constitutional aberration including partial 2p trisomy detected by classical cytogenetic studies were further evaluated using array-CGH. The patient with NB was found to have 20.5 Mb duplication (arr[hg18] 2p25.3p24.1(2,999-20,462,999)×3), while another patient had 22.3 Mb duplication (arr[hg18] 2p25.3p24.1(29,193-22,311,862)×3) at chromosome 2p together with a deletion on chromosome 18q (arr[hg18] 18q22.3q23(71,282,999-76,112,910)×1) and 17p (arr[hg18] 17p13.3(29,169-2,867,570)×1) respectively. In both cases region of gain encompassed *MYCN* genomic region but not *locus* of *ALK* gene.

**Figure 1:**
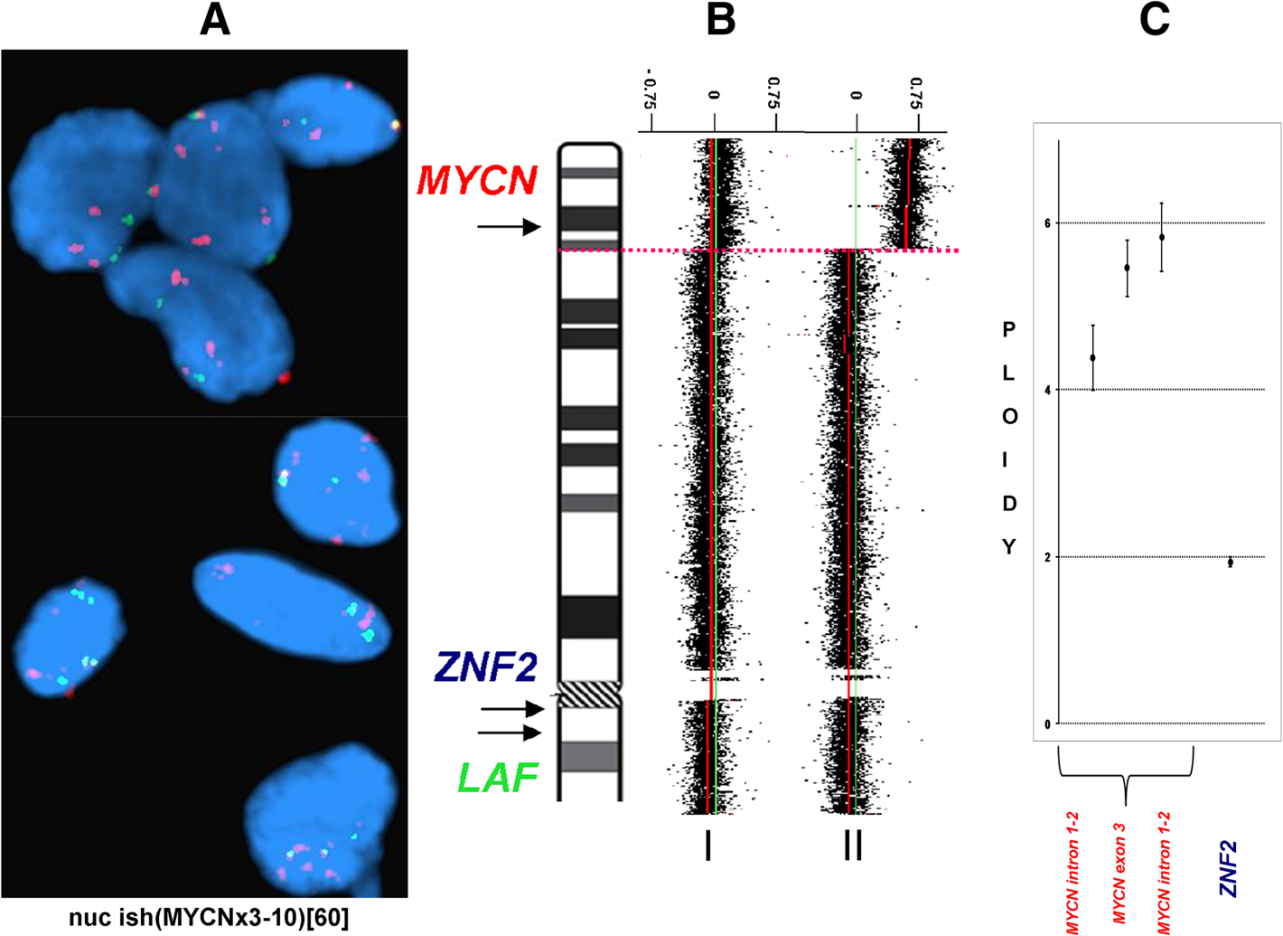
**Evaluation of *MYCN *status in the NB tumor of a patient with constitutional 2p duplication. A.** Interphase FISH analysis of the *MYCN* status in the tumor “touch imprints” (red – *MYCN*, green – *LAF* at chromosome 2q11.2). **B.** array-CGH analysis of the *MYCN* status. DNA extracted from the tumor hybridized against constitutional DNA of the same patient (I) and against a pool of control reference DNA (II). X-axis: the log2 ratio, Y-axis: chromosomal coordinates according to NCBI36/hg18. **C.** qPCR analysis of the *MYCN* status. The dosage of the *MYCN* gene (three independent qPCR amplicons) and the control gene *ZNF2* (located at chromosome 2q11.1).

Evaluation of *MYCN* status in NB tumor was performed by FISH, array-CGH and qPCR (Figure 1). Interphase FISH showed *MYCN* gain nuc ish(MYCN×3-10)[60] in the tumor cells, while evaluation of metaphase chromosomes showed no signal translocation neither to dmin nor to other chromosomes. Array-CGH showed slight elevation of the log2 ratio between the blood and the tumor and three independent qPCR experiments targeting each of the *MYCN* exons further confirmed *MYCN* gain. Besides, except for the unbalanced translocation, no other segmental chromosomal alterations or regions with loss of heterozygosity (LOH) were detected in the near-diploid tumor. *ALK* mutation status was found not-mutated both at germinal and somatic level.

**Figure 2:**
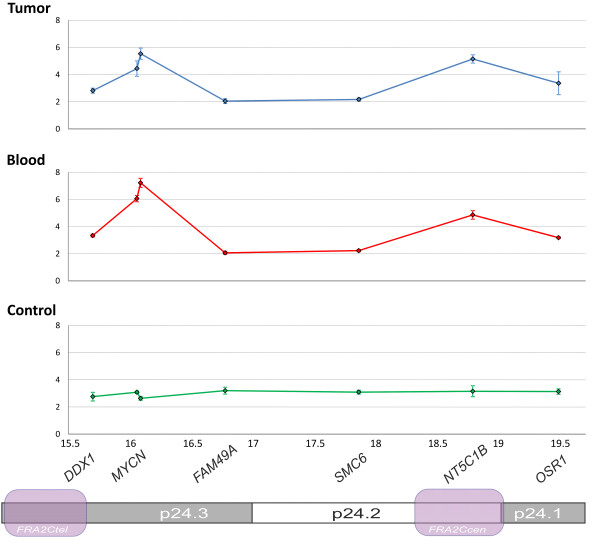
**Dosage of genes located within the *MYCN *amplicon.** Tumor - somatic (NB tumor) DNA of the patient. Blood - constitutional (peripheral blood leukocytes) DNA of the NB patient. Control - constitutional (peripheral blood leukocytes) of the control patient with constitutional partial 2p duplication but no NB. X-axis chromosomal coordinates and gene locations are presented according to NCBI36/hg18; Y-axis shows DNA ploidy. Genomic positions of the FRA2C fragile sites are given as established by Blumirch *et al*. [21].

Precise delineation of the region of *MYCN* gain within the area of duplication has been undertaken and a series of qPCR targeting the adjacent genes have been designed. As the reference, DNA from another patient with constitutional aberration leading to partial 2p trisomy, namely 46,XY,der(17)t(2;17)(p24.1;p13.3) was used. Fluctuation of the gene copy number within the 15.5-19.5 Mb region of the chromosome 2p, consistent with the boundaries of the common fragile site FRA2C has been observed both at somatic, and to lower extent also at germinal level in the NB patient. Conversely, the case without NB did not present any fluctuation of the gene copy number in the studied area (Figure 2).

## Discussion

**Figure 3:**
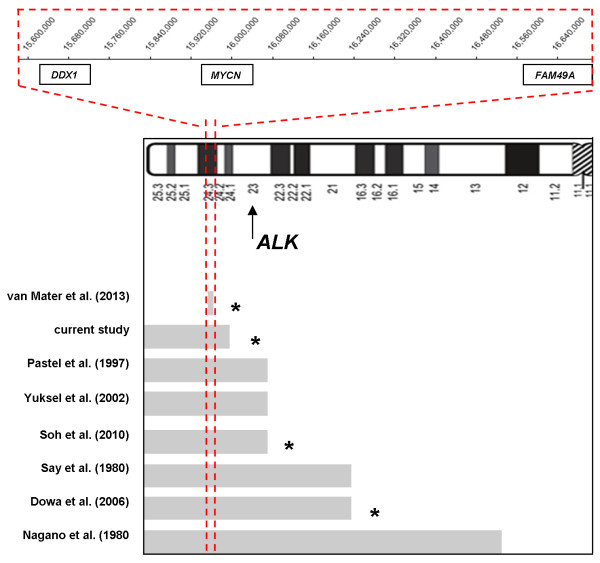
**The range of chromosome 2p aberrations in patients with constitutional partial 2p trisomy and NB.** Extend of the duplication at 2p in each of the reported patients. Note that for two cases only (van Mater *et al.* and the current study) determination of the duplicated region was performed on array-CGH level. **Red**-**dashed area**: minimal overlapping region of 1 Mbp (2:15,626,128-16,653,344), including the *locus* of *MYCN* gene. **Black asterisks**: patients who had *MYCN* status evaluated in the tumor. Chromosomal coordinates presented according to NCBI36/hg18.

The database search for the patients with congenital chromosomal aberrations who had NB retrieved seven patients with aberrations involving chromosome 2p and another seven cases with rearrangements of other chromosomes (Additional file 1: Table S1). For the patients sharing the 2p duplication, the minimal overlapping region was estimated to encompass 1.0 Mb at chromosome 2p24.1 (Figure 3). The region includes *loci* of *MYCN*, *DDX1* and partially *FAM49A* gene but not *ALK* gene. The second chromosomal region involved in the rearrangements was different in each of the 2p cases reported so far. Hence it is most likely, the monosomy of the chromosome 18q22.3-q23 does not have any relevance to NB development in the child under study, especially that none of the genes mapping to this region is considered to be a cancer-related gene.

Then again, the question arises, whether constitutional 2p trisomy could be considered an overt causative agent triggering NB development, since only a fraction (<10%) of patients with constitutional 2p duplication eventually develops NB [7-9]. To address the issue further, we have compared two patients with constitutional rearrangement at chromosome 2p involving *MYCN* but not *ALK locus*, of whom only one developed NB. We have found significant differences in gene copy number profile at *MYCN* genomic region. Areas of gains and losses at chromosome 2p24 were observed in the patient with NB (Figure 2). On somatic level, similar aberration, including *MYCN* gain, was detected in the NB sample. No other genetic event characteristic for NBs, including other segmental chromosomal aberration(s), LOHs or an *ALK* gene point mutation was found in the tumor. Metaphase FISH studies showed uneven distribution and variable degree of the gain in single cells reflecting the heterogeneous character of the tumor. Since secondary numerical and segmental chromosomal alterations are common findings in NB, this may explain why in qPCR *MYCN* copy number was lower in tumor vs. blood (Figure 2) given that the assay measures relative dosage of the genes.

The consequence of *MYCN* gain in NB remains unclear. Even though large studies did not show its prognostic significance [10] our data provide further evidence in favor of its role at early stage of NB development and tumor progression. In the light of the clinical variables: low-stage, neonatal age at diagnosis, histopathology subtype and lack of adverse genetic markers our patient should have been classified as a low-risk NB. However, the uncertainty with respect to the number of *MYCN* gene copies, eventually classified as *MYCN* gain, and the natural history of other NB patients with constitutional 2p duplication were the two rationales for choosing an intensified strategy of treatment to address the anticipated aggressive biology of the tumor. All previously published patients with partial 2p trisomy who were diagnosed with NB over the age of six months had already progressed to metastatic disease at the time of diagnosis [9,11-13]. Unfortunately, *MYCN* amplification was assessed in three patients only (Figure 3). The results were discordant: a case of a stage 1 NB was found to have 3 copies of *MYCN* on array-CGH [14]; a patient with stage 4S NB was found to have *MYCN* gain by FISH [15] and the third child with stage 4 NB was found to have *MYCN* amplification presenting as multiple double minutes (dmin) on FISH examination [9].

The molecular evaluation of the *MYCN* genomic region allowed for detection of significant differences in gene copy number pattern at the 2p24 *locus* suggestive for the presence of a cryptic complex genomic rearrangement (CGG) in our NB patient. A number of models and processes have been proposed to be involved in the origin of CGGs [16]. The ongoing debate seems to favor chromothripsis, the one-off event to be responsible for most of the structural aberrations in cancer [17]. Likewise, the recent analysis of a series of patients with constitutional CGGs has shown involvement of end-joining mechanism of double-stranded DNA breaks resembling the chromothripsis-like chromosome catastrophes in their pathogenesis [18].

Moleenar et al. [19] were the first to report presence of chromothripsis in 11% of primary NBs, on the whole in older (>1.5 years) patients with *MYCN* amplified and solely advanced stage tumors. In one of ten reported tumors, chromothripsis resulted in amplification and very strong overexpression of *MYCN*. Further evaluation of high-risk NB by means of next-generation techniques has shown no recurrent fusion transcripts and presence of substantial local rearrangements in a minority of cases only [3]. The later exclusively affected the vicinity of *MYCN locus*, with numerous complex copy number states and retention of heterozygosity in lower-copy number regions. Such a pattern of amplified/nonamplified regions within the *MYCN* amplicon was already observed in a series of NB cell lines [20]. The striking common denominator for the aforesaid NB-related CGGs and the presented case is the presence of minute areas of gains and losses in the vicinity the *MYCN* gene. Blumirch *et al.*[21] have shown that the boundaries of the CGGs involving *MYCN* detected in NBs, and also observed in the present case, cluster to the well known microhomology region: a common fragile site FRA2C on chromosome 2p24 (Figure 2).

Common fragile sites (cFS) are evolutionarily conserved AT-rich regions inherited within normal chromosomal structure well known for their relative high mutation rate [22]. When exposed to environmental factors that interfere with DNA replication the accumulation of DNA secondary structures at cFS may give rise to double-strand breaks, what in consequence may generate structural chromosomal instability [23]. Our results suggest, that the inherited unbalanced chromosomal aberration at 2p may give grounds for nonrandom clustering of chromosomal breaks at the FRA2C leading to *MYCN* amplicon formation.

## Conclusions

To our knowledge, this is the first report of a case of a constitutional genomic instability at *MYCN locus* detected in an neonate with an inherited unbalanced chromosomal rearrangement and neuroblastoma and no other genomic alteration and/or known NB risk factor. It seems that the germinal chromosomal aberration encompassing *MYCN locus* without co-involvement of *ALK* gene was sufficient to trigger cancerogenesis.

## Methods

### Patients

A patient with 46,XX,der(18)t(2;18)(p24;q23)mat presenting distinct dysmorphic features (dolichocephaly, midfacial hypoplasia, large fontanella, hypotelorism, anteverted nostrils, prominent philtrium, microretrognathia), congenital defects (ASD, PDA, cervical ribs, bilateral postaxial polidactyly) and severe neonatal hypotonia was found to have a suprarenal lesion on neonatal sonography. Further image studies (MRI and metaiodobenzylguanidine (MIBG) scintiscan) as well as bone-marrow biopsy showed presence of a single solid tumor lying in the left suprarenal area, but with no sign of metastatic lesions or bone-marrow involvement. At the age of 2 months surgery was performed. In view of the final diagnosis of locoregional Schwannian stroma-poor poorly differentiated NB (low risk according to Shimada system and INRG consensus pretreatment classification) the patient started chemotherapy according to LINES 2009 (Low and Intermediate Risk Neuroblastoma European Study) protocol, however after two courses the parents declined from further oncological treatment. Moreover, after defining maternal origin of the translocation, the family refused further genetic evaluation. The patient remains alive 2.5 years later with no sign of disease recurrence.

The second patient with 46,XY,der(17)t(2;17)(p23;p13)dn, is a mildly dysmorphic boy with multiple congenital defects including lissencephaly, cryptorchidism, duplex ureter (ureter fissus). The patient is subject to regular oncological surveillance by means of image studies and periodical testing of urine catecholamine metabolite levels. The child is alive with no sign of neoplastic process at the age of 3.8 years.

### DNA samples

DNA was isolated from the peripheral blood leukocytes of two patients with constitutional partial 2p trisomy and nine anonymous healthy female volunteers, which were utilized as the reference control DNA. Constitutional DNA samples used in array-CGH and qPCR experiments were isolated using QIAamp DNA Blood Midi Kit (Qiagen). Besides, tumor DNA, after verification of neoplastic cell content to be exceeding 80%, was extracted from a fresh-frozen neuroblastoma sample according to salting–out protocol [24].

### Karyotyping and fluorescent *in situ* hybridization

Cytogenetic studies were performed using conventional GTG–banding of lymphocyte metaphase chromosomes at a 550 band level following standard protocol. Double-color fluorescent *in situ* hybridization (FISH) was performed on interphase nuclei of NB tumor touch imprints and on lymphocytes using Cytocell N-myc Amplification Probe LPS009 for *MYCN* gene copy number detection. In each analysis at least 60 nuclei were evaluated.

### Targeted mutational analysis of *ALK* gene

Germline and somatic status of *ALK* at its mutational *hot*-*spot* (exons 23–25) was evaluated through direct sequencing (Applied Biosystem).

### Array comparative genomic hybrydization

Array – CGH analysis of patients constitutional DNA and tumor DNA was at first performed using high – resolution Human CGH 2.1 M Whole-Genome Tiling Array and later expanded using Human CGH 385 K Chromosome 2 Tiling Array and Custom CGH/LOH 37×1.4 M Arrays (NimbleGen, Roche) following the instructions provided by manufacturer. Arrays were scanned with MS200 Microarray Scanner and analyzed using NimbleScan, SignalMap and Deva v1.2.1 software (NimbleGen, Roche). All identified genomic imbalances were verified in the database of genomic variants (DGV; http://dgv.tcag.ca; last accessed May 2013).

### Quantitative real – time PCR

The results of microarray study were validated by quantitative real – time PCR (qPCR) performed on LightCycler480 System (Roche). Target genes: *DDX1*, *MYCN*, *FAM49A*, *SMC6*, *NT5C1B*, *OSR1* within the duplicated region (2p24) were assessed against a control gene *ZNF2* at 2q11.2 and two reference genes *GPR15* (3q11.2) and *ERMP1* (9p24.1). qPCR assays were performed using FastStart Universal Probe Master and specific FAM pre – labelled probes from Universal Probe Library (Roche). All samples were run in triplicates. The dosage of target genes relative to reference genes normalized to control DNA (same as reference DNA used for array-CGH study) was assessed.

## Consent

Written informed consent was obtained from the legal guardians of the patient (parents) for the publication of the data presented in the report.

## Competing interests

The authors declare that they have no competing interests.

## Authors’ contributions

BSL contributed to concept and design of the study, contributed to acquisition of data, designed the experiments, performed interpretation of data, performed database research and *in*-*silico* analyses and drafted the manuscript; MK – performed array-CGH and qPCR experiments and drafted the manuscript; JW – performed genetic evaluation and follow-up of the cases; AP – was primary oncologist responsible for anti-cancer treatment of the index case; MI – performed cytogenetical (karyotype and FISH) analyses; EIS – performed histopathological evaluation of the tumor samples and revised the draft of the manuscript critically; EAD – supervised clinical evaluation and follow-up of the cases, JL – supervised cytogenetic studies and revised the draft of the manuscript critically. All authors read and approved the final manuscript.

## Supplementary Material

Additional file 1: Table S1 Patients with constitutional unbalanced chromosomal aberrations who developed neuroblastoma. List of all patients with constitutional chromosomal aberrations who developed neuroblastoma. Click here for file
