# Supplementary material for: On the significance of germline cytogenetic rearrangements at MYCN locus in neuroblastoma
Source: Mol Cytogenet. 2013 Oct 16;6:43. doi: 10.1186/1755-8166-6-43 (PMC3819649; doi:10.1186/1755-8166-6-43)
Supplement: Additional file 1: Table S1 — Patients with constitutional unbalanced chromosomal aberrations who developed neuroblastoma. List of all patients with constitutional chromosomal aberrations who developed neuroblastoma. [file 1755-8166-6-43-S1.pdf]

Additional File 1

Patients with constitutional unbalanced chromosomal aberrations who developed neuroblastoma.

Chromosome	Neuroblastoma	Additional features	Reference
**ABERRATIONS INVOLVING CHROMOSOME 1p36**			
**46,XX,del(1)(p36.1p36.2)dn**	5 months of age; stage 4S NB successfully treated with chemotherapy; post-treatment biopsy revealed mature ganglioneuroma	dysmorphic features, developmental delay, muscular weakness, congenital heart defect (VSD)	Biegel JA, White PS, Marshall HN, Fujimori M, Zackai EH, Scher CD, Brodeur GM, Emanuel BS. Constitutional 1p36 deletion in a child with neuroblastoma. Am J Hum Genet 52 (1993) 176-82
**46,XX,del(1)(p36.33)dup(1)(p36.32p36.33)dn**	2 months of age; stage 4S NB, spontaneous regression without chemotherapy within one month, opsoclonus–myoclonus syndrome	dysmorphic features, developmental delay, seizures, corpus callosum hypoplasia	Isidor B, Le Cunff M, Boceno M, Boisseau P, Thomas C, Rival JM, David A, Le Caignec C. Complex constitutional subtelomeric 1p36.3 deletion/duplication in a mentally retarded child with neonatal neuro-blastoma. Eur J Med Genet.51 (2008) 679-84
**ABERRATIONS INVOLVING CHROMOSOME 2p**			
**46,XY,dup(2)(p21p25)**	35 days old infant, NB “in situ” no cytogenetic studies of the tumor tissue performed	Dysmorphic features, blepharo-phimosis, ectopic anus, left lung agenesis, congenital heart defect (common atrioventricular canal), seizures	Say B, Carpenter NJ, Giacoia G, Jegathesan S. Agenesis of the lung associated with a chromosome abnormality (46,XX,2p+). J Med Genet 17 (1980) 477-90
**46,XY.arr[hg18] 2p24.3(15,626,128-16,653,344)x3 dn**	11 months of age; stage 4 NB, **MYCN amplification** (dmin), 1p deletion	Dysmorphic features, developmental delay, postaxial polidactylia,	Van Mater D, Knelson EH, Kaiser-Rogers KA, Armstrong MB. Neuroblastoma in a pediatric patient with a microduplication of 2p involving the MYCN locus. Am J Med Genet 161(2013) 605-10
**46,XY,der(8)t(2;8)(p21;p23.2)mat**	1 month of age; stage 4S NB, **MYCN gain** (3-7 signals for the chromosome 2 centromere; 5-9 signals for MYCN)	Dysmorphic features, supernumerary nipples, hypospadias, congenital heart defect (double-outlet right ventricle, VSD, pulmonary stenosis), cataract due to persistent hyperplastic primary vitreous	Dowa Y, Yamamoto T, Abe Y, Kobayashi M, Hoshino R, Tanaka K, Aida N, Take H, Kato K, Tanaka Y, Ariyama J, Harada N, Matsumoto N, Kurosawa K. Congenital neuro-blastoma in a patient with partial trisomy of 2p. J Pediatr Hematol Oncol 28 (2006) 379-82

**46,XY,der(13)t(2;13)(p23;q34)dn**	17 months of age;<br>stage 4 NB<br>no cytogenetic studies of the tumor tissue performed	Dysmorphic features, postaxial polidactylia, rib deformity, seizures, skin aplasia at the posterior scalp region,	Patel JS, Pearson J, Willatt L, Andrews T, Beach R, Green A. Germline duplication of chromosome 2p and neuroblastoma. J Med Genet 34 (1997) 949-51
**46,XY,der(16)t(2;16)(p13;p11)pat**	8 months of age;<br>stage 4 NB,<br>no cytogenetic studies of the tumor tissue performed	Dysmorphic features, microophatlmos, congenital cataract, micropenis,	Nagano H, Kano Y, Kobuchi S, Kajitani T. A case of partial 2p trisomy with neuroblastoma. Jinrui Idengaku Zasshi 25 (1980) 39-45.
**46,XY,der(16)t(2;16)(p23;q13.3)**	3 months of age - stage 1 NB<br>9 months of age - stage 1 NB of the contralateral adrenal; both treated with surgery alone<br>cytogenetic studies showed 3 copies of MYCN and ALK, 17q duplication	Dysmorphic features, hypospadias, inguinal hernia, malrotation, feeding difficulties, left multi-cystic dysplastic kidney, seizures	Soh SY, Stavropoulos D, Bowdin S, Thorner P, Baruchel S, Malkin D, Meyn MS, Irwin M. Meachronous neuroblastoma in an infant with constitutional unbalanced translocation t(2;16)(p23;p13.3) involving ALK. Poster presentation at ANR Stockholm, June 21-24 2010; POC38.
**46, XX,der(17)t(2;17)(p23;q25)**	4 years of age;<br>stage 4 NB<br>no cytogenetic studies of the tumor tissue performed	Dysmorphic features, developmental delay	Yuksel A, Seven M, Karaman B, Yilmaz S, Deviren A, Hacihanefioglu S, Basaran S. Neuroblastoma in a dysmorphic girl with a partial duplication of 2p caused by an unbalanced translocation. Clin Dysmorphol 11 (2002) 39-42
**46, XX, der(18)t(2;18)(p24;q23)mat**<br>**arr[hg18] 2p25.3p24.1(2,999-20,462,999)x3, 18q22.3q23(71,282,999-76,112,910)x1**	7 day of life, stage 2 NB<br>**MYCN gain**<br>(for details refer to the main text)	Dysmorphic features, postaxial polidactylia, cervical ribs, congenital heart defects (ASD, PDA)	Current study
**OTHER CHROMOSOMAL REARRANGMENTS**			
**46,XX,der(7)t(7;8)(q34;p12)mat**	congenital NB in an aborted fetus under 24 weeks of gestation	dysmorphic features, cleft lip and palate, congenital heart defect (aortic stenosis, hypoplastic left heart), annular pancreas, gall bladder aplasia	Fryns JP, Petit P, Moerman F. Cassiman JJ, van den Berghe H. 8p trisomy in a malformed foetus. Ann Genet. 25 (1982) 162-3.
**46,XY,der(10)t(10;15)(q26;q22)pat**	congenital NB in an aborted fetus under 24 weeks of gestation	dysmorphic features, congenital heart defect (coarctation of the aorta ASD/VSD); agenesis corpus callosum, supernumerary kidneys, intestinal malrotation	Fryns JP, Moerman P, Kleczkowska A, van den Berghe H. Neuroblastoma and partial 15q duplication in the fetus. H Ann Genet. 37 (1994) 42-3.

**46,XX,der(10)t(3;10)(q21;q26)dn**	congenital NB in an aborted fetus under 24 weeks of gestation	dysmorphic features, congenital heart defect (VSD), lung hypoplasia, cystic hygroma of neck, hydronephrosis, Potter sequence	Qureshi F, Jacques SM, Johnson MP, Reichler A, Evans MI. Microscopic neuroblastoma in a fetus with a de novo unbalanced translocation 3;10. Am J Med Genet. 53 (1994) 24-8
**46,XY,der(13)t(13;15)(q34;q23)mat**	14 months of age, stage 4 NB	dysmorphic features, craniosynostosis, club feet varus, agenesis corpus callosum	Sanger WG, Howe J, Fordyce R, Purtilo DT. Inherited partial trisomy #15 complicated by neuroblastoma Cancer Genet Cytogenet. 11 (1984) 153-9.
**46,XY,der(11)t(11;12)(q23;q23)**	No data	dysmorphic features, developmental delay,	Koiffmann CP, Gonzalez CH, Vianna-Morgante AM, Kim CA, Odone-Filho V, Wajntal A. Neuroblastoma in a boy with MCA/MR syndrome, deletion 11q, and duplication 12q. Am J Med Genet 58 (1995) 46-9
